# A Novel Forming Method of Traditional Chinese Medicine Dispersible Tablets to Achieve Rapid Disintegration Based on the Powder Modification Principle

**DOI:** 10.1038/s41598-018-28734-x

**Published:** 2018-07-09

**Authors:** Pan Li, Bi Feng, Hong Jiang, Xue Han, Zhenfeng Wu, Yaqi Wang, Junzhi Lin, Yi Zhang, Ming Yang, Li Han, Dingkun Zhang

**Affiliations:** 1 College of Pharmacy, Chengdu University of Traditional Chinese Medicine, Key Laboratory of Systematic Research, Development and Utilization of Chinese Medicine Resources in Sichuan Province—Key Laboratory Breeding Base of Co-founded by Sichuan Province and MOST, Chengdu, P. R. China; 2 Key Laboratory of Modern Preparation of TCM, Ministry of Education, Jiangxi University of Traditional Chinese Medicine, Nanchang, P. R. China; 3 Teaching Hospital of Chengdu University of Traditional Chinese Medicine, Chengdu, P. R. China; 4 Chengdu Institutes of Food and Drug Control, Chengdu, P. R. China

## Abstract

Slow disintegration and poor solubility are common problems facing the dispersible tablets of Traditional Chinese Medicine (TCM). In an early study, the research group found that co-grinding of extracts and silica could achieve a rapid disintegration effect, though the mechanism of this effect was not thoroughly elucidated. In this study, Yuanhu Zhitong dispersible tablets (YZDT) were selected as a model drug to explore the mechanism of rapid disintegration and dissolution. First, eight types of silica were used to prepare modified YZDT, and their disintegration time and amount of dissolution within 5 min were measured. Next, the powder properties of eight types of silica were investigated. By correlation analysis, it was found that the average pore size and density of silica were closely related to the effect of promoting disintegration. It was determined that the co-grinding of silica and extracts provided high porosity for the raw material drug, and its abundant narrow channels provided a strong static pressure for water penetration to achieve a rapid disintegration effect. Meanwhile, it was found that the addition of silica had a certain effect on promoting dissolution. Our results provide a highly operational approach for improving the disintegration and dissolution of TCM dispersible tablets. Meanwhile, this approach is also beneficial for establishing a high-quality evaluation index for silica.

## Introduction

Traditional Chinese Medicine (TCM) or natural medicine is becoming increasingly popular for its advantages of multi-ingredients, multi-links and multi-targets. However, many active ingredients isolated from Chinese herbs have poor water solubility, including the anti-tumor substance taxinol^1^, the anti-inflammatory substance andrographolide^2^, and the analgesic substance tetrahydropalmatine^3^. It is thus important to achieve rapid release and rapid onset in the process of preparation design. Dispersible tablets are typical fast release formulations, which can disintegrate rapidly and disperse evenly in water. The tablets have high bioavailability because of these features. Furthermore, the production process is simple, does not require special machines, and becomes an important carrier of product development in pharmaceutical enterprises^4–6^. In the early 1980s, aspirin and other two dispersible tablets were included in British pharmacopoeia^7^. In the mid-1990s, Chinese pharmacists attempted to combine Chinese herbal extracts with dispersible tablet formulations and successfully developed Salvia miltiorrhiza dispersible tablets, Yuanhu Zhitong dispersible tablets (YZDT), and andrographolide dispersible tablets. Currently, there are fifty-seven TCM dispersible tablets on the market^8–10^.

However, this combination is not simple because of the significant difference between physical properties and dosages of the raw materials. For most chemical drugs, their physical properties are single, and the dosages are small^11,12^, while the physical properties of Chinese herbal extracts are complex, and the dosages reach 30–40%^13^. Therefore, the preparation demands of dispersible tablets cannot not be met by only optimization of composition and proportion of disintegrating agents. Because researchers have ignored the complex physical and chemical properties of Chinese herbal extracts, a series of technical problems have been encountered, such as disintegration retardation, loose quality, and tablet splitting. These problems have seriously plagued the production of Chinese patent medicine and have increased the cost of production and application^14^.

Different from the crystalline structures of chemical medicine^15^, Chinese herbal extracts usually present as a paste or amorphous powder. The extracts are not typical insoluble substances for mixing with a large number of polysaccharides and other hydrophilic ingredients^16^. These macromolecular organic materials accumulate and expand upon absorption, which forms a high viscosity water barrier on the surface of tablets, which prevents the water from rapidly permeating from surface to the interior. Only when the extracts are fully swelled and dissolved and the cohesion between macromolecular organic matter decreases are the permeable channels of water molecules opened. This mechanism explains the slow disintegration of TCM dispersible tablets.

Previously, our research team attempted to change the physical properties of Chinese herbal extracts to solve the problem of disintegration retardation, referring to the powder surface modification technology in materials science. It was found that the co-grinding of extracts and a small quantity of silica could achieve a rapid disintegration effect and obtain positive results in 10 types of TCM dispersible tablets. If silica dioxide was used only as filler and not ground with extracts, an ideal speed collapse effect could not be achieved^17–19^. These results demonstrated that the effect of silica on dispersible tablets was achieved by the modification of extract properties. In addition, silica was inexpensive and easy to obtain, and the price was less than 10% of super disintegrating agent, which endowed this method with a strong application value.

**Figure 1:**
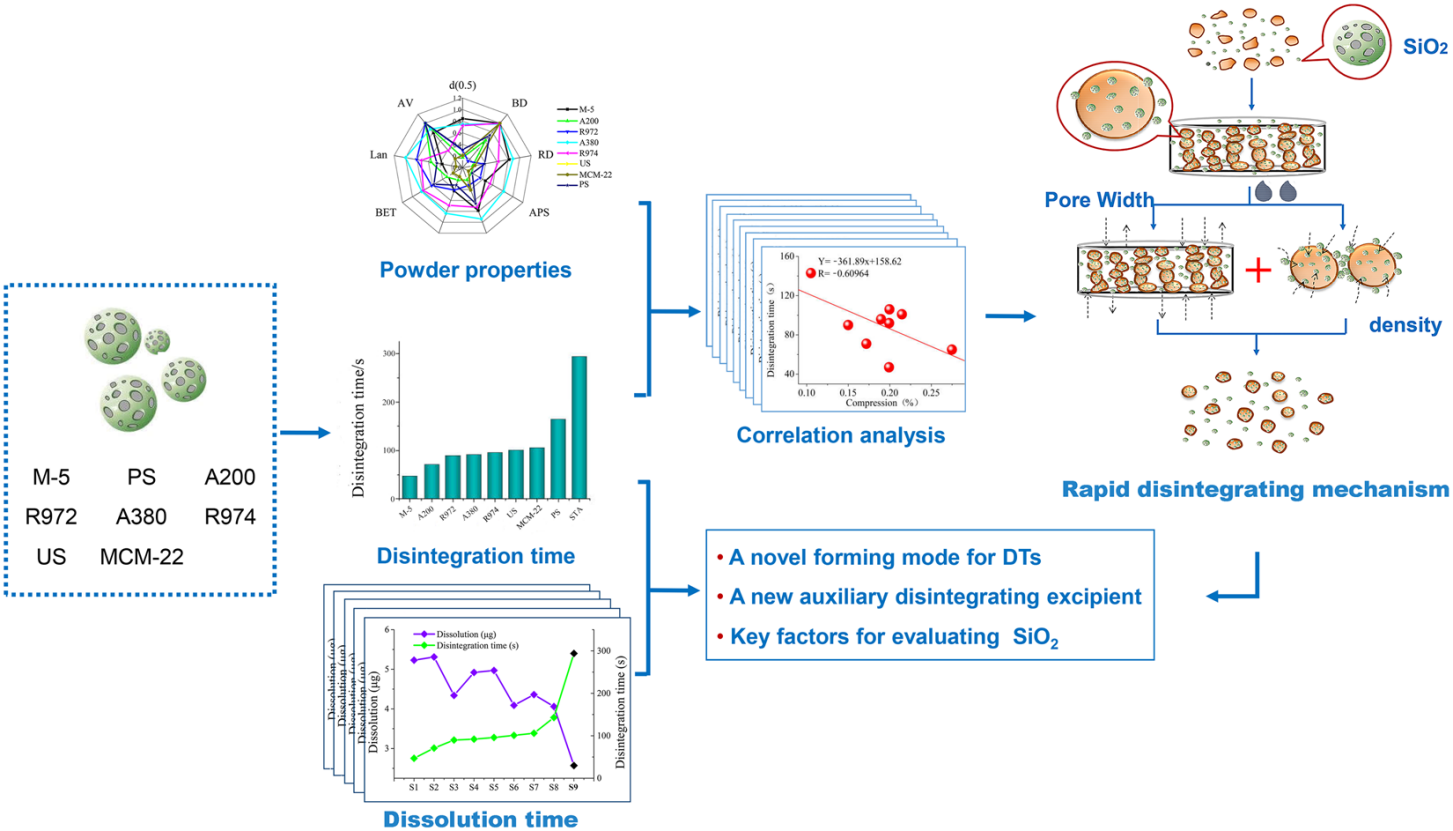
The experimental flow chart.

However, the principle of co-grinding of silica with the extract to promote rapid disintegration of TCM dispersible tablets was not clear. It was necessary to further study the correlation between the powder properties of silica and the disintegration effect. Furthermore, there were many types of pharmaceutical silica, including colloidal silica, silica gel, carbon-white, hydrophobic colloidal silica, and mesoporous silica^20–23^. It was worth studying whether all types of silica could achieve this effect. Moreover, whether the rapid disintegration of dispersible tablets would promote the immediate release of ingredients also needed to be explored. In this manuscript, a famous Chinese patent medicine with analgesic effect, YZDT, was selected as a model drug. Initially, a series of powder properties and water absorption of eight types of commercial silica were analyzed. Next, correlation analysis was used to explore the main powder properties affecting the disintegration effect. On this basis, we further studied the release ability of five active components in YZDT after silica modification (Fig. 1). In general, our research attempts to reveal the key factors of silica that promote disintegration and drug release of dispersible tablets. It will be beneficial to form a new pattern for the preparation of TCM dispersible tablets, which is of great interest to pharmacists.

## Results

### Quality control

A total of 31 components were identified, which are shown in Table 1.

**Table 1:** MS/MS data in the positive and negative ESI modes and the identification results of YZDT.

t_R_/min	[M+H]^+^/[M+H]^−^ (m/z)	ppm+/ppm^−^	MS/MS	name	formula
9.32	356.1872/−	2.81/−	340.1543, 192.1010, 177.0798	Corybulbine	C_21_H_25_NO_4_
10.78	305.1014/−	−3.61/−	203.0335, 245.0969	Oxypeucedanin	C_16_H_16_O_6_
9.47	370.1998/−	−5.40/−	324.1259, 352.1559	α-Allocryptopinea	C_22_H_27_NO_4_
7.83	342.1723/−	5.26/−	178.0858, 151.1365, 326.1365	Tetrahydrojatrorrhizinea	C_20_H_23_NO_4_
10.77	203.0335/−	−4.43/−	147.0426, 107.9675	Xanthotoxol/bergaptol	C_11_H_6_O_4_
9.19	339.1438/−	−9.46/−	188.0729, 149.0590	Jatrorrhizinea	C_20_H_20_NO_4_^+^
9.28	370.1683/−	7.83/−	294.1130, 306.1136	Cryptopine	C_21_H_23_NO_5_
8.39	356.1872/−	2.81/−	340.1543, 239.1506	D-Glaucine	C_21_H_25_NO_4_
9.88	321.1015/−	4.36/−	250.0835, 276.1030, 292.0955, 264.0997	Coptisinea	C_19_H_14_NO_4_^+^
10.1	354.1706/−	0.28/−	190.0873, 165.0910, 336.1213	Dehydrocorybulbine	C_21_H_22_NO_4_^+^
10.42	352.1559/−	2.84/−	336.1205	N-Methylcanadine	C_21_H_21_NO_4_
10.78	305.1014/−	−3.61/−	203.0335	Oxypeucedanin hydrate	C_16_H_16_O_6_
9.87	337.1310/−	−1.17/−	276.1013, 149.0509, 176.0712, 292.0955	Berberinea	C_20_H_18_NO_4_^+^
10.25	335.1130/−	−0.30/−	121.0658	Byakangelicina	C_17_H_18_O_7_
10.96	367.1749/−	−9.26/−	350.1392, 366.1720, 322.1436	Dehydrocorydalinea	C_22_H_24_NO_4_^+^
9.34	356.1872/−	2.81/−	294.1236	Tetrahydropalmatinea	C_21_H_25_NO_4_
10.09	370.1998/−	−5.40/−	165.0927, 192.1010, 354.1706	Corydalinea	C_22_H_27_NO_4_
13.30	271.0978/−	2.95/−	171.0444, 227.0338, 211.0761, 241.0491	Alloisoimperatorin	C_16_H_14_O_4_
9.48	324.1259/−	7.10/−	149.0590, 176.0712, 206.1178	Tetrahydrocoptisine	C_19_H_17_NO_4_
13.23	231.1003/−	−7.79/−	147.0450, 175.0410	Demethylsuberosin	C_14_H_14_O_3_
13.7	271.0945/−	−9.22/−	245.0969	isoimperatorin	C_16_H_14_O_4_
8.02	356.1872/−	2.81/−	340.1573, 163.0643, 151.0741	Glaucine	C_21_H_25_NO_4_
9.93	340.1543/−	−1.76/−	149.0590	columbamine	C_20_H_20_NO_4_^+^
12.81	+/352.1175	−/2.84	320.0905, 324.1222, 334.1025, 336.0800	Protopinea	C_20_H_19_NO_5_
11.03	+/231.0288	−/2.60	143.0485, 187.0810, 203.0335	5-Methoxy-8-hydroxy-psoralen	C_12_H_8_O_5_
9.19	+/338.1385	−/2.36	250.0867, 278.0817, 280.0983, 294.1130, 306.1100, 322.1069	Nantenine	C_20_H_21_NO_4_
8.10	324.1222/−	−4.32/−	192.1010, 294.0779	L-tetrahydrocoptisine	C_19_H_17_NO_4_
13.88	301.1085/−	2.98/−	105.0327, 215.0330, 233.0452, 301.1085	Cnidilin	C_17_H_16_O_5_
13.52	187.0390/−	−2.67/−	131.0493, 159.0476	Psoralen	C_11_H_6_O_3_
13.03	+/385.1250	+/9.87	231.0288, 283.1712, 339.2305	Sen-byakangelicol	C_21_H_22_O_7_
9.03	409.1493/−	−1.22/−	163.0617, 179.0920	Nodakenin	C_20_H_24_O_9_

### Results of disintegration test

The hardness of the prepared tablets was 40 N. The disintegration results of 9 tablets are shown in Table 2. It was shown that M-5 had the best auxiliary disintegration effect while STA (plain film, S9) took the longest time to disintegrate the tablets. The modification of silica can significantly reduce the disintegration time of YZDT, and different silica had different disintegration effects. Therefore, exploring how the different types of silica promote disintegration is the key step for inducing TCM dispersible tablets to disperse.

**Table 2:** Disintegration time of eight kinds of modified YZDT.

SiO_2_	T(s)	SiO_2_	T(s)	SiO_2_	T(s)
S1	47 ± 0.54*	S4	92 ± 0.52*	S7	106 ± 0.46*
S2	71 ± 0.53*	S5	96 ± 0.11*	S8	143 ± 0.84*
S3	90 ± 0.75*	S6	101 ± 0.91*	S9	294 ± 0.50

Versus control group (S9), *p < 0.05.

### Powder properties of eight types of silica

In Table 3, d(0.5) indicates the powder that particle size was accounted for 50% of the total^24^. The particle size of M-5 was the smallest, while US had the minimum value. M-5 had the minimum bulk density (BD), real density (RD), and average pore size (APS), while precipitated silica (PS) had the maximal BD, RD, APS and minimum compression rate (COM). The specific surface area of hydrophilic silica (M-5, A200, A380) was significantly smaller than other types. It can be concluded from Table 3 that the angle of repose (AR) was variable among all silicas, which led to differences in flowability. MCM-22 was the worst, and the best was M-5. It could be concluded that silica produced by different manufacturers exhibited different powder properties.

**Table 3:** Results of powder properties of eight kinds of silica.

SiO_2_	d (0.5) μm	BD (g/mL)	RD (g/mL)	APS (Å)	AR (°)	COM (%)	BET (m^2^/g)	Langmuir (m^2^/g)	AV (cm^3^/g)
S1	14.019	0.036	0.045	87.083	33.74	0.199	186.832	258.619	0.407
S2	13.495	0.038	0.045	105.517	39.62	0.172	190.055	263.914	0.501
S3	7.519	0.040	0.047	100.471	36.80	0.150	99.4863	144.983	0.250
S4	13.002	0.046	0.058	93.270	42.24	0.199	366.759	502.367	0.855
S5	7.055	0.048	0.059	118.896	37.67	0.190	140.261	201.800	0.417
S6	4.772	0.064	0.082	178.385	36.80	0.215	167.186	229.208	0.746
S7	5.972	0.115	0.143	233.172	45.00	0.200	100.889	141.328	0.588
S8	12.960	0.232	0.259	289.495	39.08	0.105	180.028	248.097	1.303

**Figure 2:**
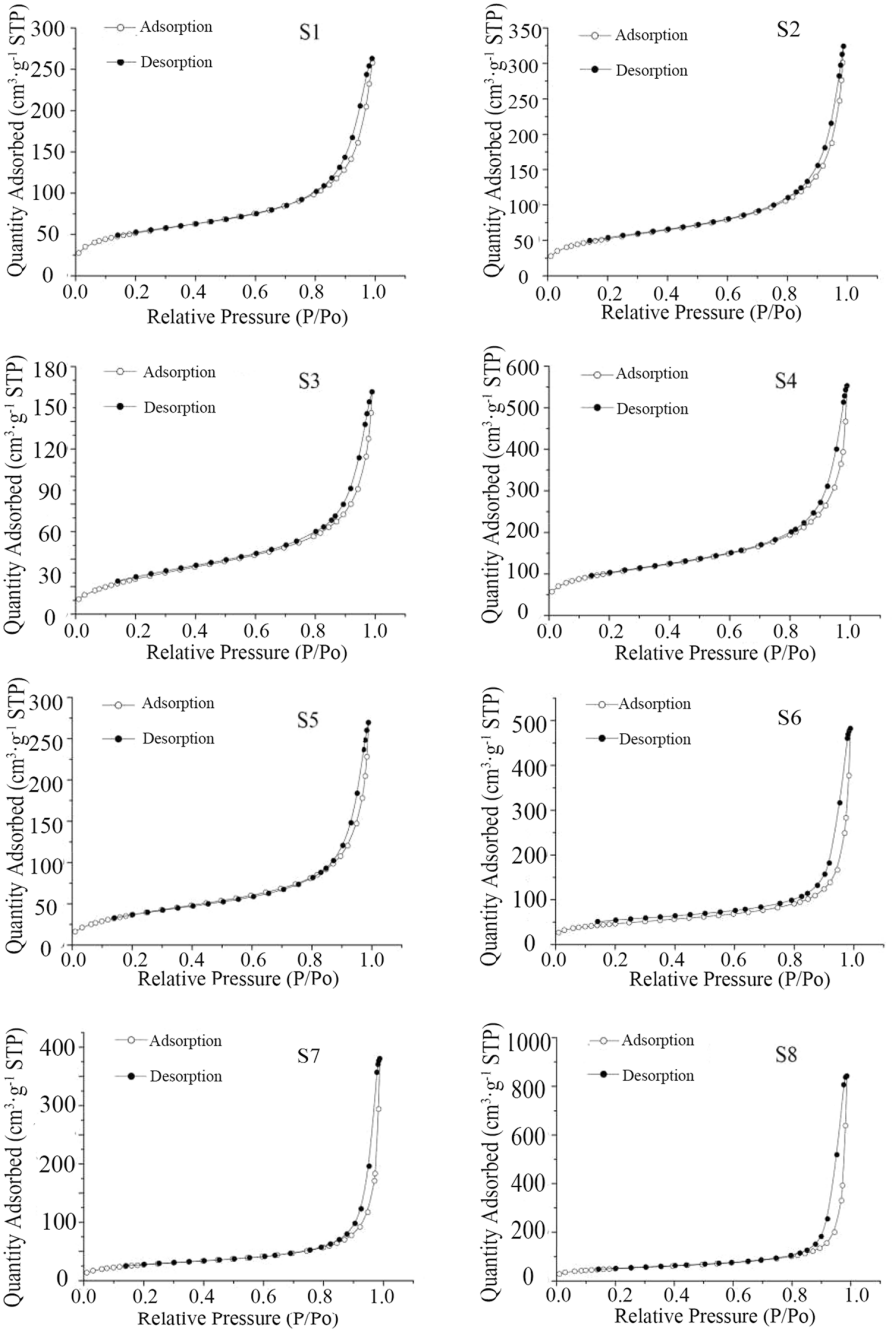
N_2_ adsorption desorption isotherms of eight kinds of silica.

Figure 2 shows that the isotherms of eight types of silica belong to Type IV adsorption isotherms. The typical characteristics of nitrogen adsorption curves are that the adsorption-desorption curves suddenly increase in the high-pressure zone, where the P/P_0_ value is approximately 0.8~1, and the hysteresis loop appears. These phenomena are related to the capillary condensation phenomenon of mesoporous materials (diameter was between 2 and 50 nm). The conclusion could be drawn from the above results that the silica was a type of fine powder with a microporous structure.

### Results of the dissolution test

**Figure 3:**
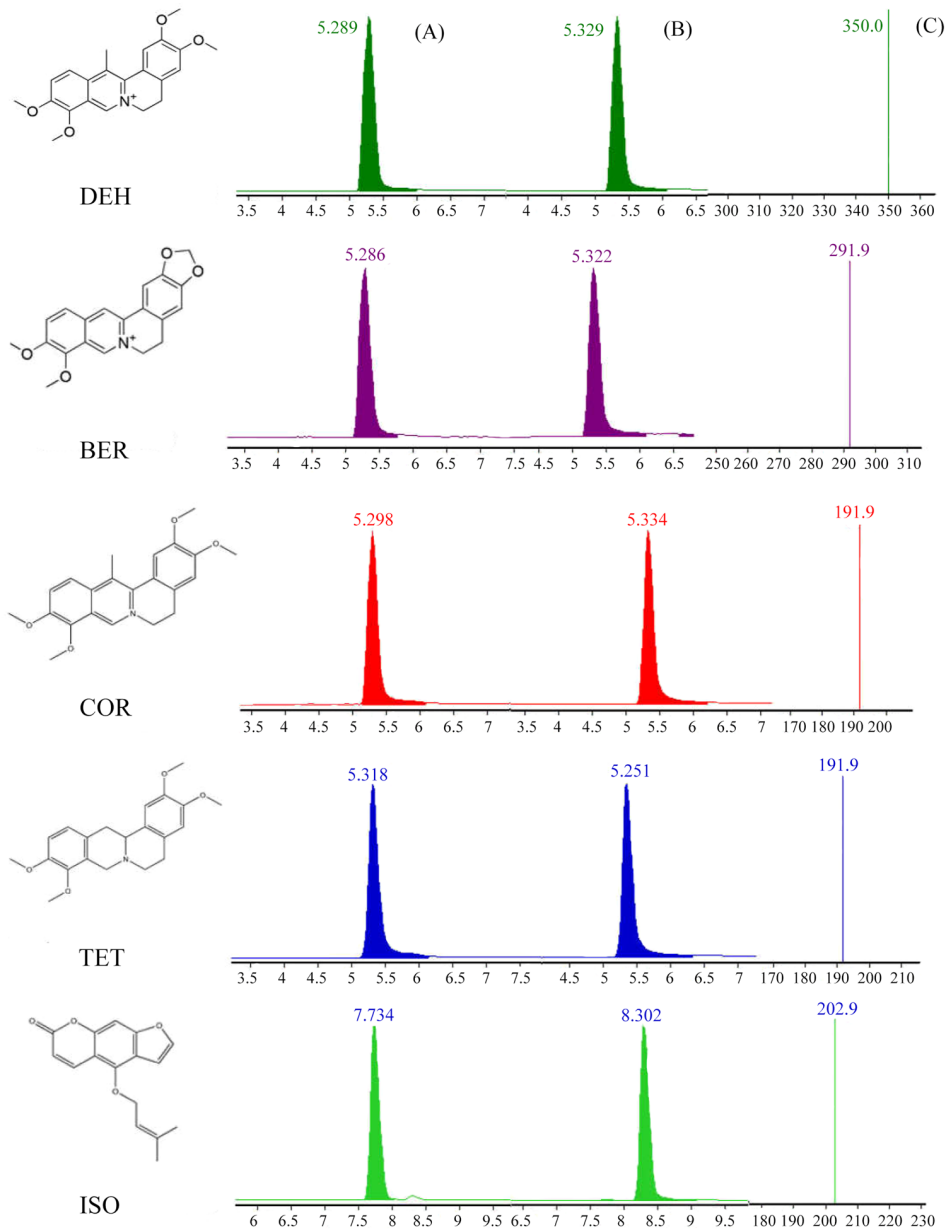
MRM chromatograms of sample (**A**) and reference substances (**B**) and second mass spectrum of reference substances (**C**).

The MRM chromatogram of five components (dehydrocorydaline (DEH), berberine (BER), corydaline (COR), tetrahydropalmatine (TET), isoimperatorin (ISO) are shown in Fig. 3, and the content of the five components is listed in Table 4. These results showed that almost all of the five components dissolved relatively faster from A 200 modified YZDT, while the PS was slower. It was more clear that the amount of dissolution in STA was considerably lower than other silica modified samples, even less than 50%. This finding indicated that the addition of silica played an important role in the dissolution of active components.

**Table 4:** The dissolution of five components in eight kinds of dispersible tablets at 5 min.

SiO_2_	TET(μg)	BER(μg)	ISO(μg)	COR(μg)	DEH(μg)
S1	23.82 ± 1.78*	1.65 ± 0.12*	5.23 ± 0.34*	7.25 ± 0.49*	1.56 ± 0.24*
S2	27.51 ± 0.31*	1.79 ± 0.04*	5.31 ± 0.02*	7.44 ± 0.04*	2.89 ± 0.11*
S3	21.70 ± 1.75*	1.52 ± 0.13*	4.34 ± 0.43*	5.76 ± 0.60*	1.83 ± 0.16*
S4	25.34 ± 0.76*	1.57 ± 0.07*	4.92 ± 0.10*	6.76 ± 0.15*	2.80 ± 0.14*
S5	27.89 ± 0.40*	1.87 ± 0.05*	4.97 ± 0.06*	6.75 ± 0.02*	2.75 ± 0.06*
S6	22.70 ± 1.43*	1.52 ± 0.11*	4.09 ± 0.26*	5.59 ± 0.39*	2.18 ± 0.10*
S7	23.15 ± 1.31*	1.61 ± 0.10*	4.36 ± 0.18*	6.11 ± 0.27*	2.13 ± 0.12*
S8	22.19 ± 1.22*	1.45 ± 0.08*	4.06 ± 0.19*	5.69 ± 0.27*	1.84 ± 0.09*
S9	11.10 ± 0.74	0.82 ± 0.06	2.57 ± 0.12	3.70 ± 0.17	0.85 ± 0.09

Versus control group (S9), *p < 0.05.

### Correlation Analysis Test

**Figure 4:**
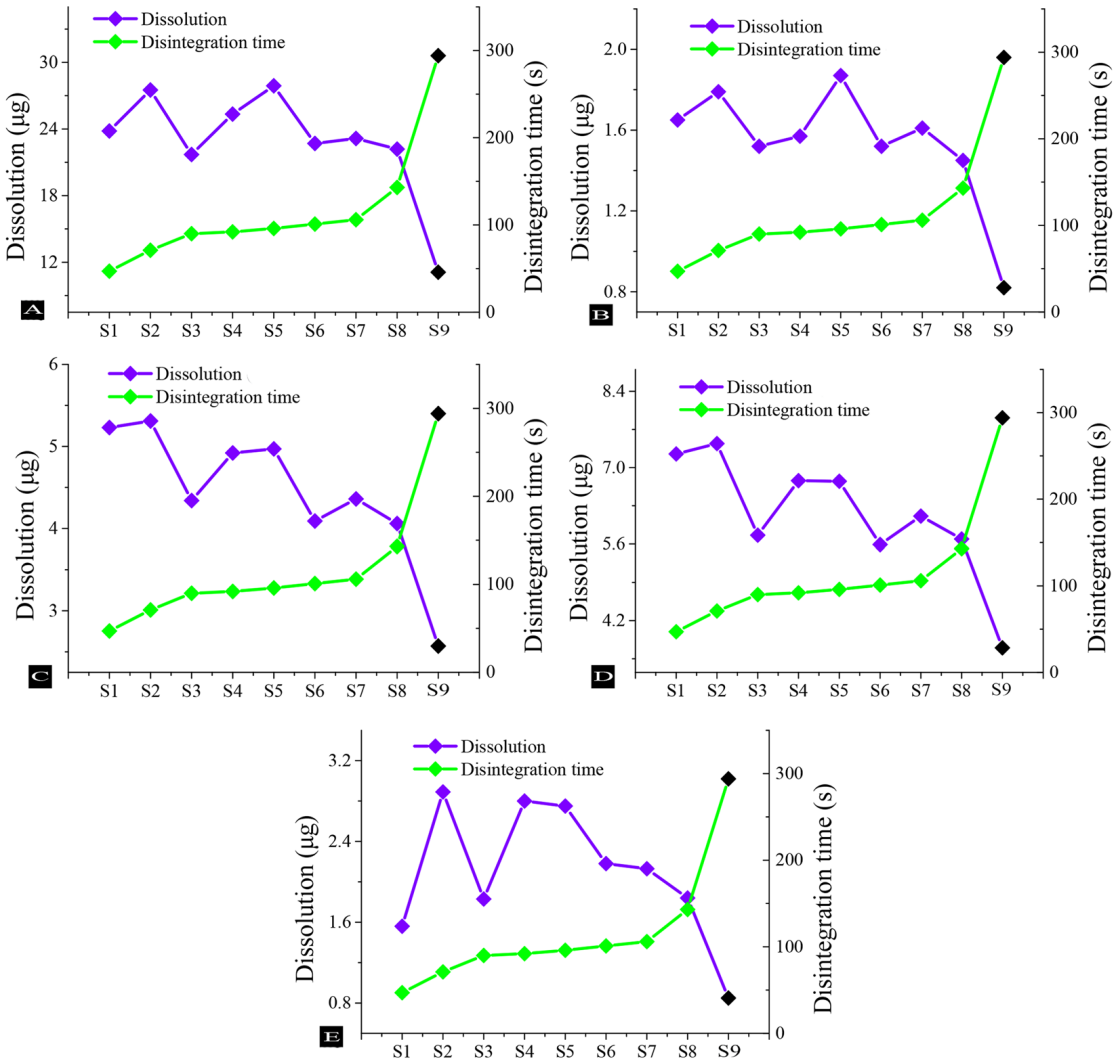
Disintegration and dissolution of dehydrocorydaline (**A**), berberine (**B**), corydaline (**C**), tetrahydropalmatine (**D**), isoimperatorin (**E**).

The disintegration and dissolution data were analyzed, as shown in Fig. 4. As the disintegration time increased, the content of dissolution generally showed a gradually decreasing trend. Clearly, Fig. 4 shows that the disintegration of the STA was the slowest, and the dissolution effect was the most unsatisfactory. Therefore, it could be concluded that the promotion of disintegration could also be conducive to the dissolution of active components.

**Figure 5:**
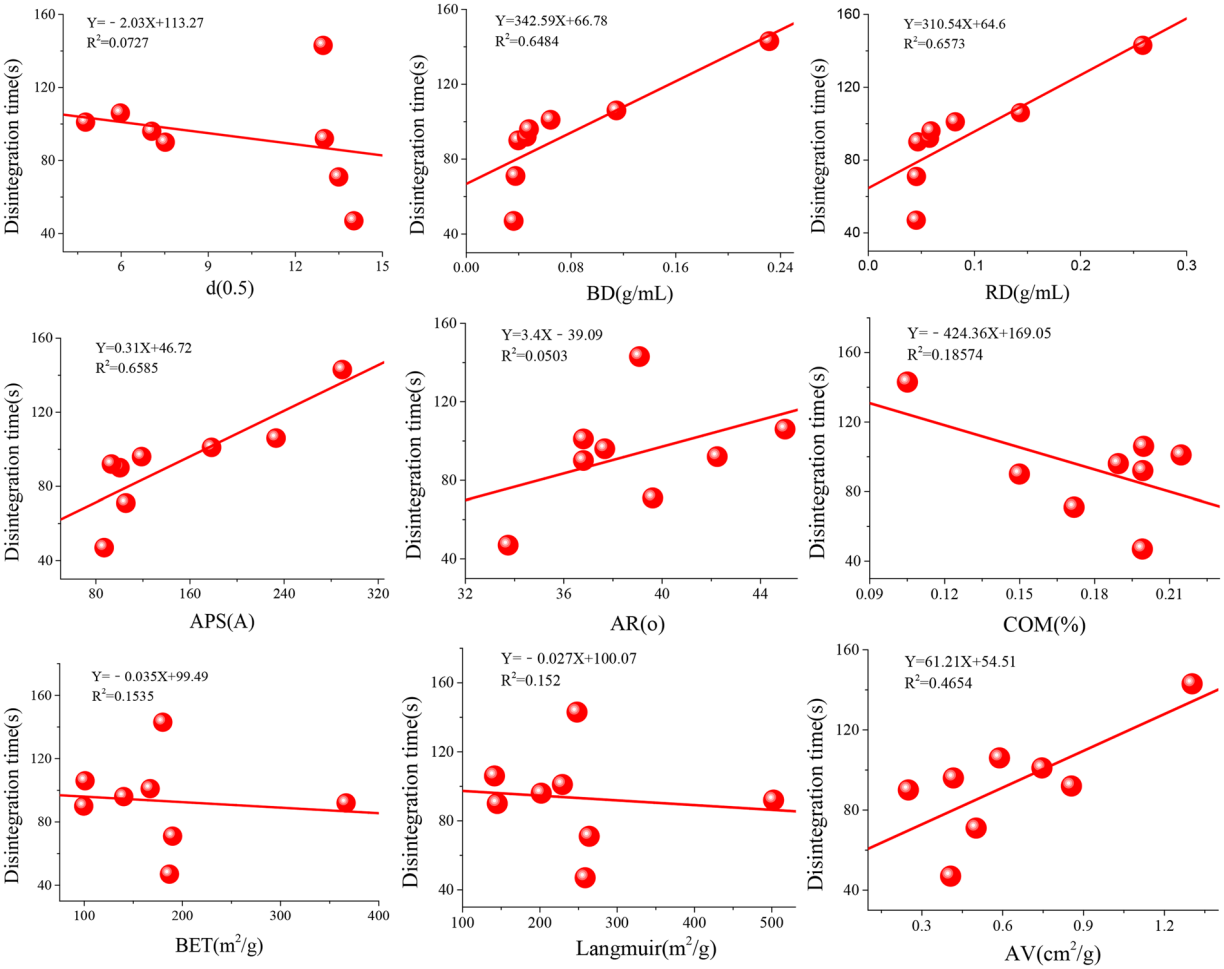
Correlation between YZDT disintegration time and powder properties of silica.

To explore the micromechanism of silica in promoting the disintegration and drug release of dispersible tablets, the research team analyzed the correlation between disintegration time and powder properties. The analytical method was simple linear correlation and was performed using Origin 9.0 software. The correlation analysis results are shown in Fig. 5. According to the R value, it was clear that d(0.5), AR, CS, BET, and Langmuir were almost not correlated with disintegration time, and the highest one was APS with the R value of 0.6785. The R value of BD and RD also reached 0.6484 and 0.6573. These results suggested that the aiding effect of silica on YZDT might be related to its APS and density.

### Results of water absorption

**Figure 6:**
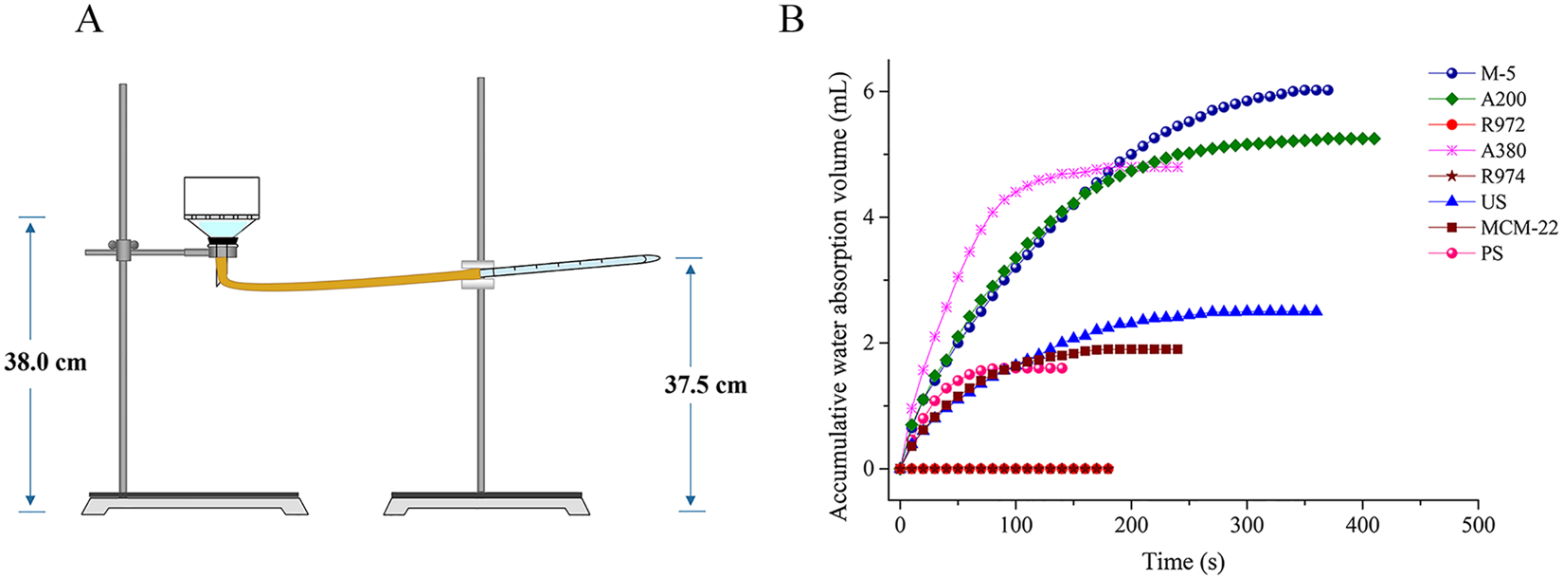
Water desorption device (**A**), accumulative water absorption volume of eight kinds of silicas (**B**).

The water absorption device is shown in Fig. 6A. The water absorption data of eight kinds of silica was analyzed by Origin 9.0 software, as shown in Fig. 6B. In Fig. 6B, M-5 and A200 had a strong water absorption capacity, while US, MCM-22 and PS showed weak effects. In addition, R972 and R974 are hydrophobic silicas and showed no absorption of water. There was a certain relationship between water absorption and promotion of disintegration effect. The stronger the water absorption was, the shorter the disintegration time was.

## Discussion

**Figure 7:**
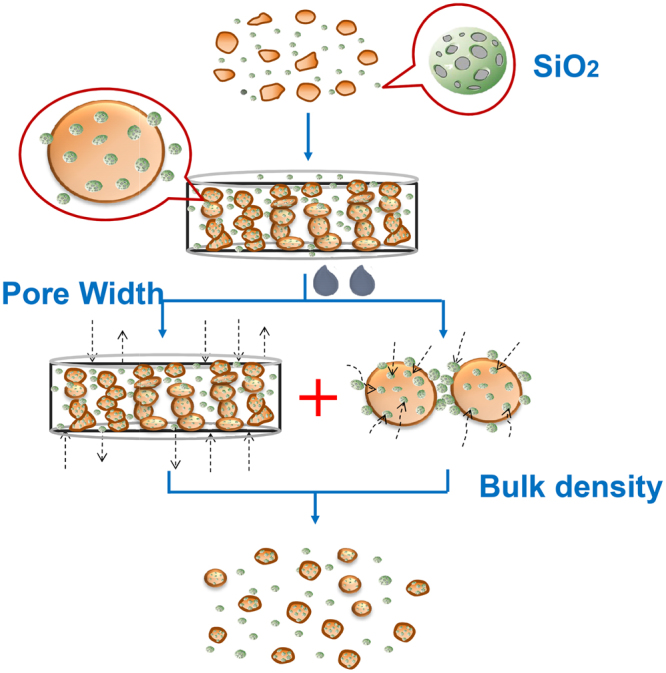
Rapid disintegrating mechanism.

According to our research, APS and density have been demonstrated to be the key factors affecting the rapid disintegration of dispersible tablets. The smaller APS and density were, the stronger the disintegration performance was. The mechanism of this phenomenon is shown in Fig. 7. The co-grinding of silica and extracts provided high porosity for the raw material drug, and its rich narrow channel provided a strong static pressure for water penetration across a high viscosity water barrier on the surface of the tablets^25–27^. Based on the Laplace equation, the static pressure of water is inversely proportional to the APS. The smaller the APS was, the stronger the water absorption power was, and the faster the water absorption was, which was also the microscopic mechanism by which the silica channels affected the disintegration of the dispersible tablets. Meanwhile, the density of silica also had a great effect on rapid disintegration. The lower the density was, the higher the quantity of silica particles per unit mass was, and the more able the particles were to fully contact and disperse with the extracts, which was conducive to reducing the cohesion of the extracts and forming a micro-environment that is beneficial to disintegration. Meanwhile, the addition of silica also served to reduce the use of a portion of the disintegrants. On the one hand, the elastic recovery was effectively controlled; on the other hand, the cheap silica could reduce production costs. This study formed a new technology for the preparation of dispersible tablets such that the rapid disintegration of TCM dispersible tablets was no longer dependent upon the role of disintegrants, greatly simplifying the difficulty of screening excipients.

As a medicinal excipient, the quality evaluation indexes of silica were various in different countries’ pharmacopoeia, primarily including pH, chloride, sulfate, iron salt, heavy metal, ash, moisture, content and other indicators. These indicators effectively ensure the purity and safety of the silica. However, due to different sources and manufacturers, the silica was made by different production methods and different process parameters, which led to discrepant physical properties. It was unreasonable to measure the application of silica as a pharmaceutical excipient with these indicators alone. Therefore, a high-quality evaluation index of silica needed to be established. The macroscopic nature was closely related to the microstructure. In this paper, we showed that the APS and density were the key indicators for evaluating silica as an auxiliary disintegrant. The establishment of the standard range on APS and density could effectively determine the auxiliary disintegration function, which was the significant value of this study.

According to the Laplace equation, the smaller the APS of the silica was, the stronger the water absorption was. At the same time, the water absorption of modified YZDT was verified in this paper.

## Conclusion

Silica is a type of auxiliary disintegrant with strong water absorption, nonexpansion and high security. The average pore size and density were the key influencing factors to evaluate disintegrative performance. In addition, the co-grinding of silica and extracts could significantly speed the disintegration of dispersible tablets. This approach was conducive to the dissolution of active components. Meanwhile, the cost of production was somewhat reduced. This approach will be beneficial to form a new pattern for the preparation of TCM dispersible tablets.

## Materials and Methods

### Materials

Cabosil M-5 was acquired from Cabot Co. (M-5, USA, S1). Aerosil A200 (A200, No. 972234, S2), Aerosil R972 (R972, No. 99033975, S3), Aerosil A380 (A380, No. 99033911, S4), Aerosil R974 (R974, No. 972345, S5) were purchased from Evonik Industries AG (Essen, Germany). Ultrafine silica (US, No. 20160923, S6), Precipitated silica (PS, No. 20160918, S8) were acquired from Shanghai Beimo Industrial Co., Ltd. (Shanghai, China). Molecular sieve MCM-22 (S7) was obtained from Tianjin Yuanli Chemical Co., Ltd. (Tianjin, China). Angelica (Angelicae Dahuricae Radix, No, 1701056), Vinegar Corydalis (Vinegar Corydalis Rhizoma, No, 1609025) were acquired from Sichuan New Lotus Chinese Herbal Medicine Co, Ltd(Chengdu, China). CMS-Na of pharmaceutical grade (No. 140901) was obtained from Anhui Shanhe Medicinal Materials Co., Ltd. (Anhui, China). PVPP of pharmaceutical grade (No. 050916002) was obtained from Chongqing Tektronix Meier Material Technology Co., Ltd. (Chongqing, China). MCC of pharmaceutical grade (No. 20140906) was purchased from Shandong Liaocheng Awa Pharmaceutical Co, Ltd. (Shandong, China). Standards of dehydrocorydaline (DEH, No. CHB170220), berberine (BER, CHB160408), corydaline (COR, No. CHB160814), tetrahydropalmatine (TET, No. CHB150730), isoimperatorin (ISO, No. CHB160917) were purchased from Chroma-Biotechnology Co., Ltd. (Chengdu, China). The purity of all compounds is more than 98%. In addition to chromatographic grade methanol, all other chemicals used were of analytical grade.

### Preparation and evaluation of dispersible tablets

#### Preparation of extracts

Angelica and Vinegar Corydalis were respectively crushed into coarse powder by HX-200 (Yongkang Xi’an Hardware Factory, China). Next, 588 g of Vinegar Corydalis coarse powder and 445 g of Angelica coarse powder were extracted twice with 5-fold volume of ethanol with a purity of 60% for 1 h each. The extracts was filtered through a qualitative filter paper to yield the sample solution. Next, the filtrate was concentrated at 81 °C followed by freeze-drying, and grinding to powder^17^.

#### Preparation of YZDT

To study the correlation between the properties of silica and disintegration time of dispersible tablets, 5% silica was ground with extracts until fully modified. At the same time, 5% soluble starch was used as the control group. Then, CMS-Na (80 g), MCC (80 g) and PVPP (80 g) were accurately weighed to make granules and 1000 tablets were pressed by DP30A single punching (Beijing Gylongli Sci &Tech Ltd., China).

#### Identification of Material base

First-order mass spectrometry was carried out to determine the chemical substances in YZDT, and the related information of pseudo-molecule-ion peak ([M]^+^, [M+H]^+^, [M−H]^−^) was obtained. In the corresponding mode, pseudo-molecule-ions were specified as the parent ion to start second-order fragment-MS determination. According to the structural information of second-order mass spectrometry, chemical components in YZDT were identified.

#### Measurement of disintegration time

According to the method of scattered uniformity in part IV, Ch. P 2015^28^, the disintegration time of YZDT was measured. Each sample was measured three times^14^.

#### Powder properties of silica

The powder properties of silica were determined, including particle size distribution, specific surface area and porosity, density and compression and angle of repose.

#### Particle size distribution

A little sample was placed on the sample groove, and the Scirocco 2000 dry dispersion method was adopted to determine the distribution of particle size by Marvern MS2000 laser particle size analyzer (Marvern Ltd, USA)^24^.

#### Specific surface area and porosity

The powder was added to a hemisphere of the sample tube and placed in a Smart Prep065 pretreatment system with N_2_ blowing to constant mass under heating at 200 °C. Next, the BET method was adopted to measure specific surface area and porosity and was tested at 55 points of each sample by TriStar 3000 automated surface area and porosity analyzer (Micromeritics Ltd., USA)^29,30^.

#### Density and compression

Sample was added to a 20 mL cylinder, and the volume was approximately 10 mL. At the same time, precision volume (V1) and weight (m1) were recorded. Loose density (ρ1) was calculated as formula (1). Next, the cylinder was beaten 60 times per minute for 5 minutes. In addition, the vibratory volume (V_2_) was recorded to figure out vibratory density (ρ_2_, listed in formula 2). Compressibility (COM) was the ability to reduce the volume of powder under pressure (listed in formula 3).

1 $${\rho }_{1}=\frac{{m}_{1}}{{V}_{1}}$$

2 $${\rho }_{2}=\frac{{m}_{1}}{{V}_{2}}$$

3 $$C{\rm{o}}m=\frac{({\rho }_{2}-{\rho }_{1})}{{\rho }_{2}}$$

#### Angle of repose

The double funnel method was used to measure the angle of repose in closed and independent conditions. The radius (r) and height (h) of cone were accurately measured. The angle of repose (θ) was calculated as formula (4).

4 $$\tan (\theta )=\frac{h}{r}$$

#### MS Conditions

Samples were analyzed by an Agilent 1260 high-performance liquid chromatograph and Agilent 6460C triple-quadrupole tandem mass spectrometer (Agilent Technologies, Santa Clara, CA, USA) using an Agilent Technologies Zorbax Eclipse XDB-C18 column (4.6 mm × 150 mm, 5 μm). The column temperature was 35 °C and 10 μL of the sample solution was injected into the system. The mobile phase was composed of (A) 0.1% aqueous formic acid in water and (B) acetonitrile using a gradient program of 100–60% B for 0~1 min, 60–10% B for 1~3 min, 10–0% B for 3~5 min, and 0–100% B for 5~10 min with a mobile flow rate of 1 mL/min^31^.

Mass spectrometric scans were obtained by electrospray ionization (ESI) in positive-ion mode with a scanning interval 100–1000 m/z. The main parameters for MS were set as follows: gas temperature, 300 °C; gas flow, 11 L/min; nebulizer, 35 psig; capillary voltage, 4000 V; atomizer pressure 15 psi (1 psi = 6.895Kpa). MS parameters and MRM transitions of each analyte are shown in Table 5.

**Table 5:** The detected ion pairs of five components.

components	RMM	ISM	PI	DI	FV	CE
TET	355.2	ESI±	356.1	191.9	145	27
BER	336.1	ESI±	336.1	291.9	148	30
ISO	270.1	ESI±	271.0	202.9	73	5
COR	369.2	ESI±	370.1	191.9	150	29
DEH	366.2	ESI±	366.2	350.0	140	30

#### Measurement of dissolution

The dissolution study was performed by placing the YZDT in 200 mL of ultra-pure water that had been treated by ultrasound for 30 minutes, using the small cup method at 100 rpm and 37 ± 0.5 °C. Dissolution medium (2 mL) was withdrawn at 5, 10, 20, 30 and 40 min through a 0.22 μm microporous membrane^32^. The sample was analyzed by LC-MS (Agilent 1260 high performance liquid chromatography, Agilent, USA; Agilent 6460 C Triple Quadrupole LC/MS with ESI ion source, Agilent, USA), each YZDT was paralleled six times and five components were determined.

#### Correlation analysis between disintegration time and powder properties

Horizontal axis showed the powder properties of each silica, and vertical axis showed the results of disintegration time. The simple correlation analysis was adopted to test the correlation coefficients using Origin 9.0 software. The larger the correlation coefficient R value was, the greater the influence of powder properties was.

#### Water absorption capacity verification

The water absorption device consists of a sintered glass funnel and a 10 mL pipette. The funnel and pipette were connected by a rubber tube to control the sintered glass funnel, which was 38 cm away from the table, 37.5 cm from the head of the pipette to the table, meaning that the pipette was filled with water and the funnel filter plate was fully wetted without water leakage. A filter paper with a small opening in the middle was placed in the sintered glass funnel, and a hollow glass tube with smooth inner circular wall and a diameter of 2.7 cm was placed under the opening. A certain amount of silica was poured into the hollow glass tube and stacked into a loose powder heap with the same thickness and diameter. At the same time, the change of the liquid level in the pipette was captured by a digital camera. The temperature was controlled at 18~22 °C and the humidity was 40~60%. When the water was saturated, the record was stopped. The video was introduced into the computer to read the data of liquid surface changing with time. Each sample was repeated 3 times^14^.

#### Statistical analysis

All the values were expressed as means ± SD. The results were analyzed by one-way analysis of variance (ANOVA) using SPSS22.0 software (SPSS Inc., USA). *p* < 0.05 was considered to be statistically significant.
